# Supplementary material for: Disordered Protein Tail Is Wagging Poly(ADP-ribosyl)ation
Source: Int J Mol Sci. 2025 Aug 22;26(17):8166. doi: 10.3390/ijms26178166 (PMC12427752; doi:10.3390/ijms26178166)
Supplement: Supplementary file 1 [file ijms-26-08166-s001.zip › Suppemental Data.pdf]

SUPPLEMENTARY APPENDIX FOR

Disordered Protein Tail is Wagging Poly(ADP-ribosyl)ation

Authors: Guillaume Bordet[1], Yaroslava Karpova[1], Saraynia Espeseth[1], Gavin Mitzel[1], Zachary Bigelow[1], and Alexei V. Tulin[1]*

Affiliations:

1 - University of North Dakota, Grand Forks, ND

*Corresponding Author.

THIS FILE INCLUDES:

Supplemental Figure S1. PARG proteins exhibit disordered tails.

Supplemental Figure S2. PARG disordered tails are highly divergent across species.

Supplemental Figure S3: Uncropped Blots Corresponding to Figure 2 and Figure 4.

Supplemental Figure S4: PARG-WT Localization in Diploid Tissues.

Supplemental Figure S5: The absence of PARG disordered tail region leads to developmental delays.

Supplemental Figure S6: PARG-Cat Localization in Diploid Tissues.

Supplemental Figure S7: Only the Higher Molecular Weight Form of PARG-Cat Is Detected in the Nucleus.

Supplemental Figure S8: PARG-D-tail Localization in Diploid Tissues.

Supplemental Figure S9: Predicted PARG SUMOylation Motifs are Located on Catalytic Domain and on the Disordered Tail Region.

Supplemental Movie S1 legend: The Disordered Tails of Human and Drosophila PARG Are Structurally Distinct.

Supplemental Table S1 legend: Mass Spectrometry Data.

Supplemental Table S2 legend: Proteins Interacting with the Catalytic Domain.

Supplemental Table S3 legend: Proteins Interacting with the Disordered Tail Region legend.

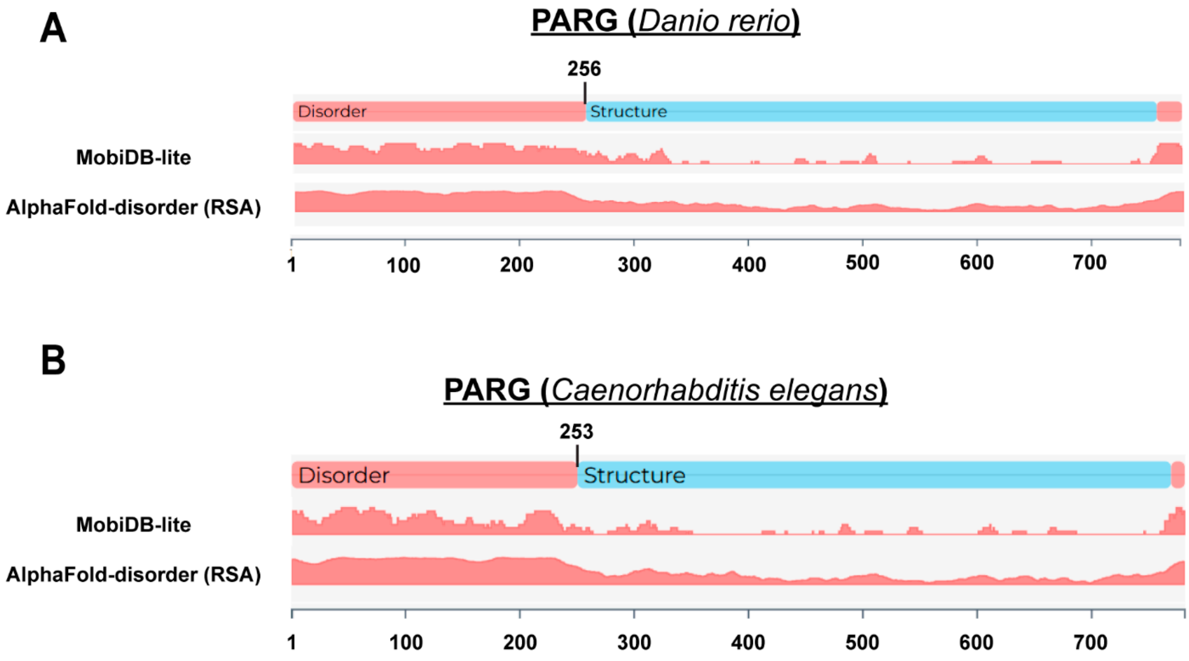

Supplemental Figure S1. PARG proteins exhibit disordered tails. (A–B) Structural disorder predictions using MobiDB-lite and AlphaFold indicate that the N-terminal tails of *Danio rerio* PARG-a (A) and *Caenorhabditis elegans* PARG-1 (B) are intrinsically disordered. Disordered regions (highlighted in red) span residues 1–256 in *D. rerio* and 1–253 in *C. elegans*, while structured regions are shown in blue. These disordered N-terminal tails support the conserved presence of intrinsically disordered regions (IDRs) across species.

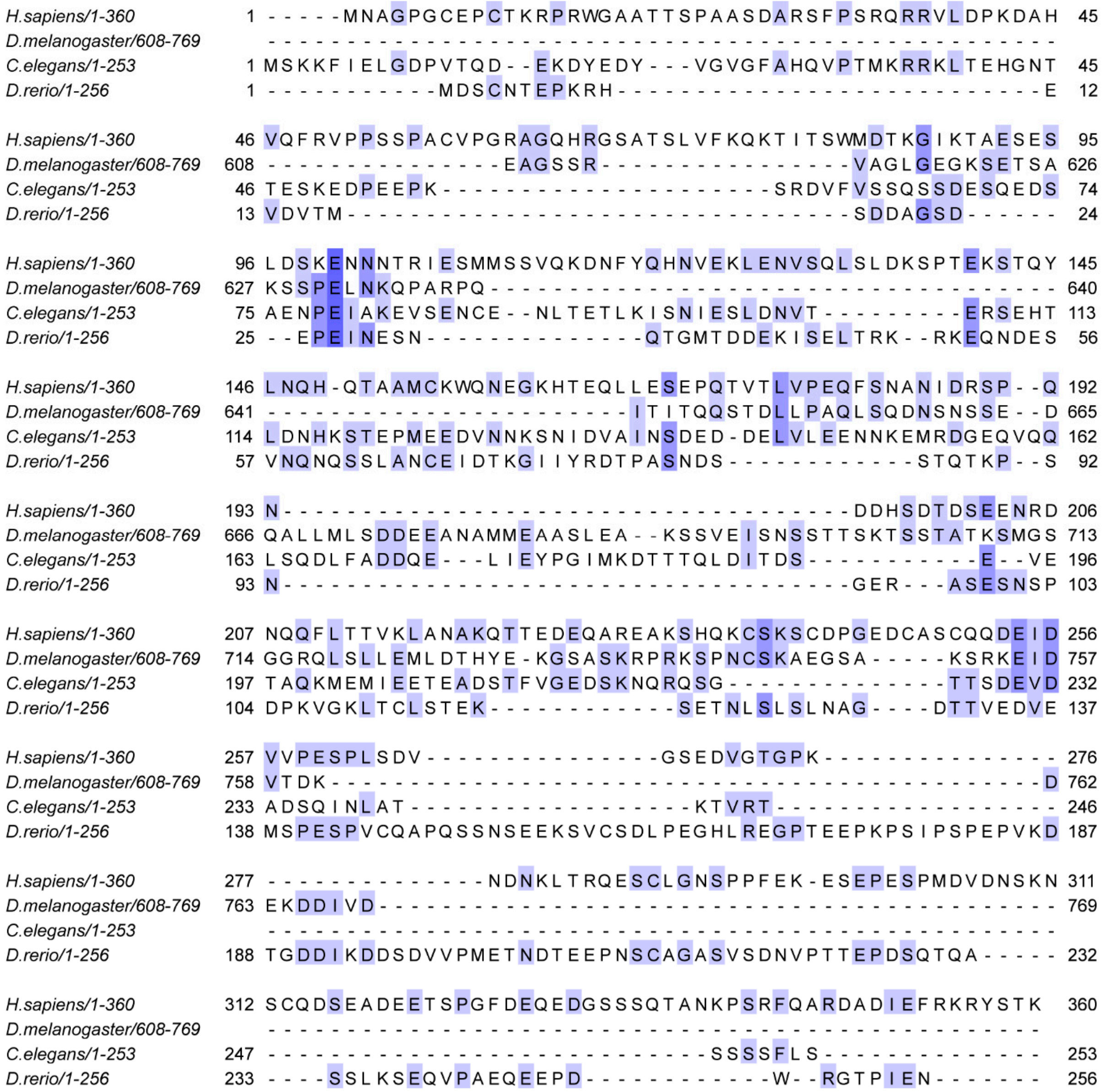

Supplemental Figure S2. PARG disordered tails are highly divergent across species. Sequence alignment of the intrinsically disordered tails of PARG proteins from four species: residues 1–360 of *Homo sapiens* PARG, 608–769 of *Drosophila melanogaster* PARG, 1–253 of *Caenorhabditis elegans* PARG, and 1–256 of *Danio rerio* PARG. Alignments were performed using Clustal Omega with default parameters. The blue color gradient indicates sequence identity, highlighting the lack of conservation among disordered tail regions across species.

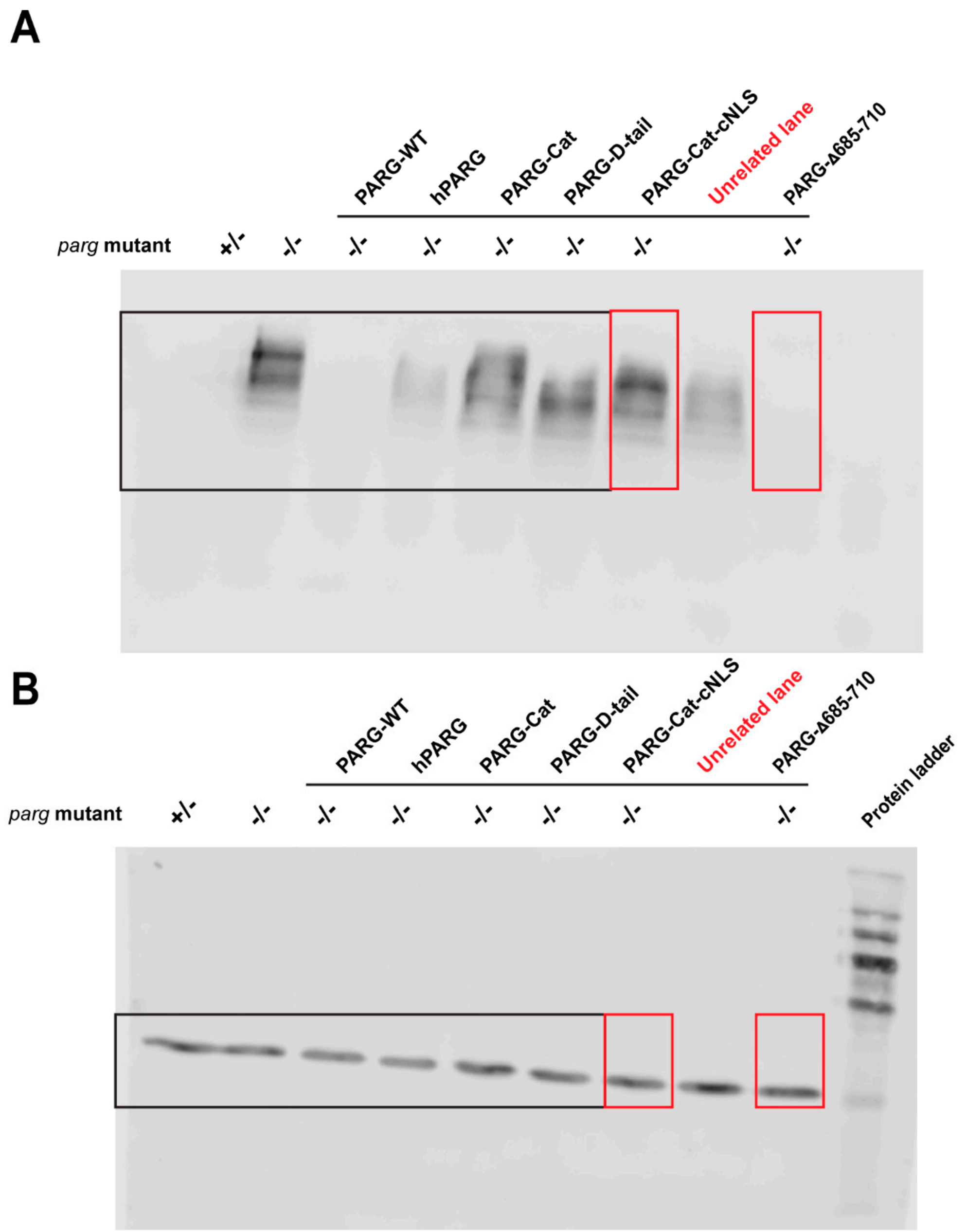

Supplemental Figure S3. Uncropped Blots Corresponding to Figure 2 and Figure 4. A-B) Uncropped western blots for the top panel (A) and bottom panel (B) of Figure 2A (regions indicated by black boxes) and Figure 4D (regions indicated by red boxes). The lane outlined in red contains a sample unrelated to this study. The last lane contains the molecular weight ladder.

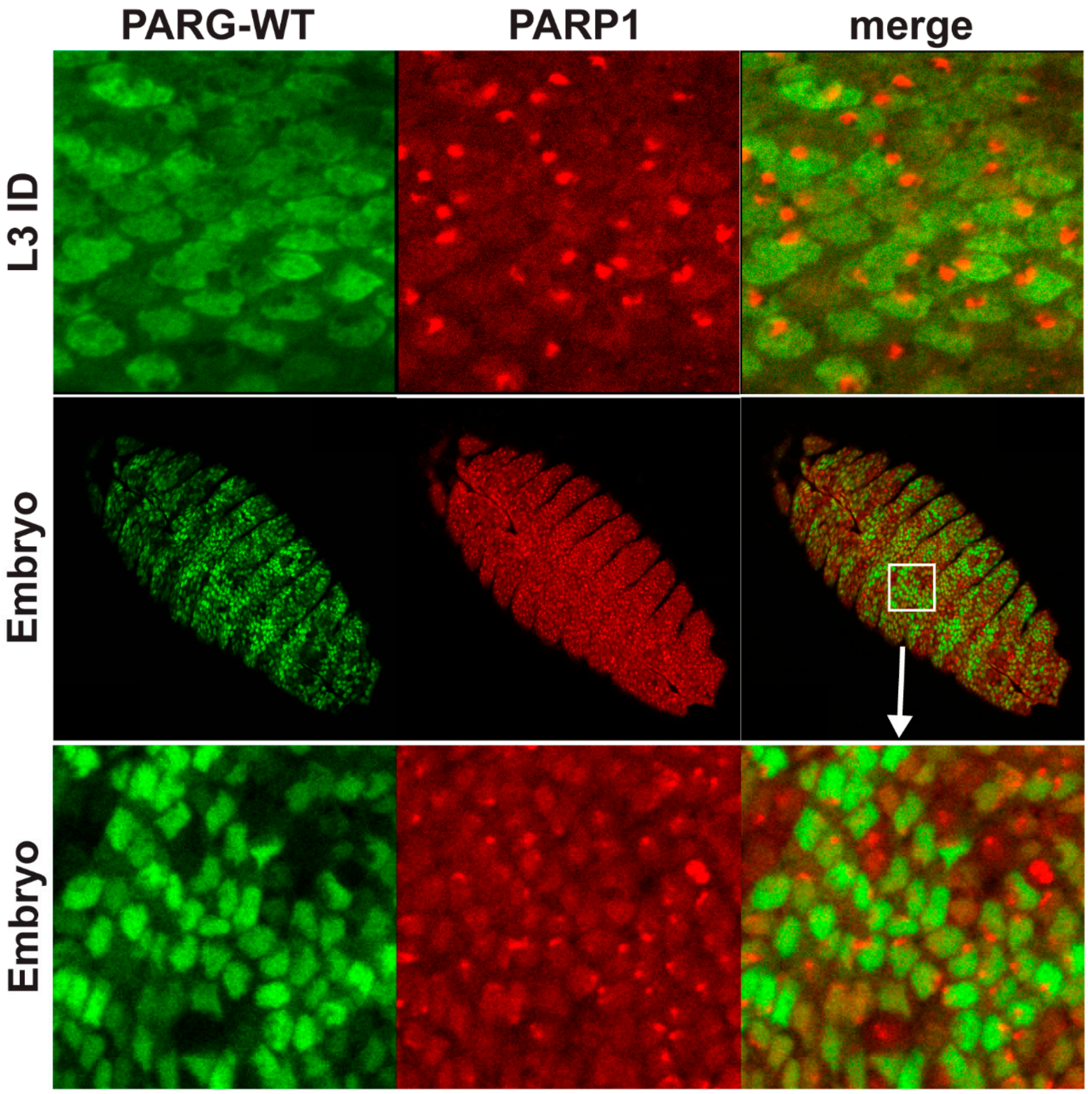

Supplemental Figure S4. PARG-WT Localization in Diploid Tissues. Representative images showing the localization of PARG-WT (left panels) and PARP1 localization (middle panels) in diploid tissues, including third instar larval imaginal discs, and embryonic tissues.

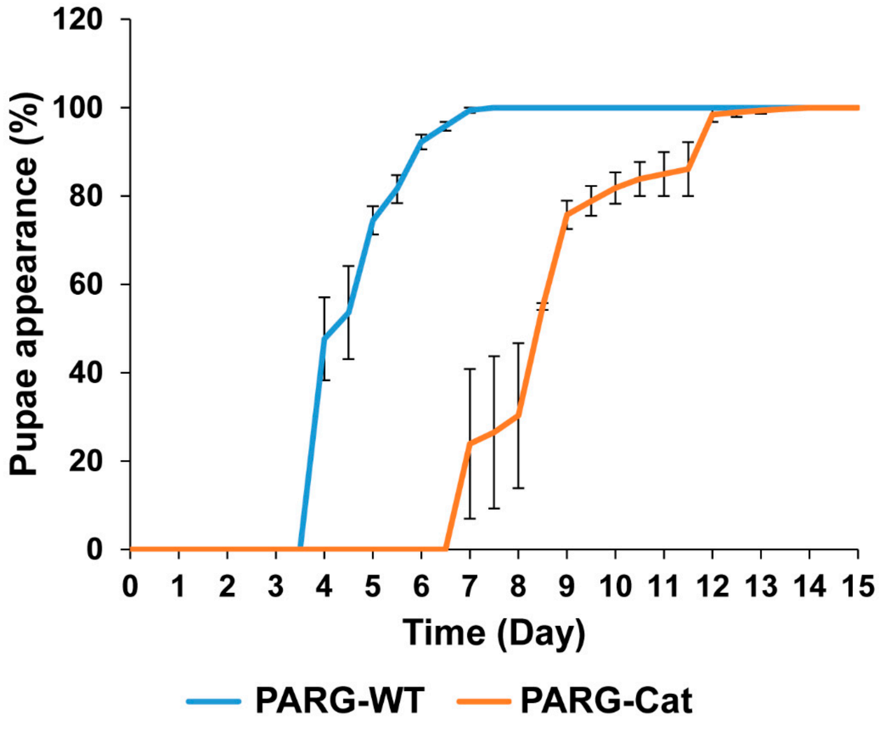

Supplemental Figure S5. The absence of PARG disordered tail region leads to developmental delays. The timing of pupae appearance normalized by the total number of pupae in each bottle (see Materials & Methods). Error bar represents Standard Error of Mean (SEM).

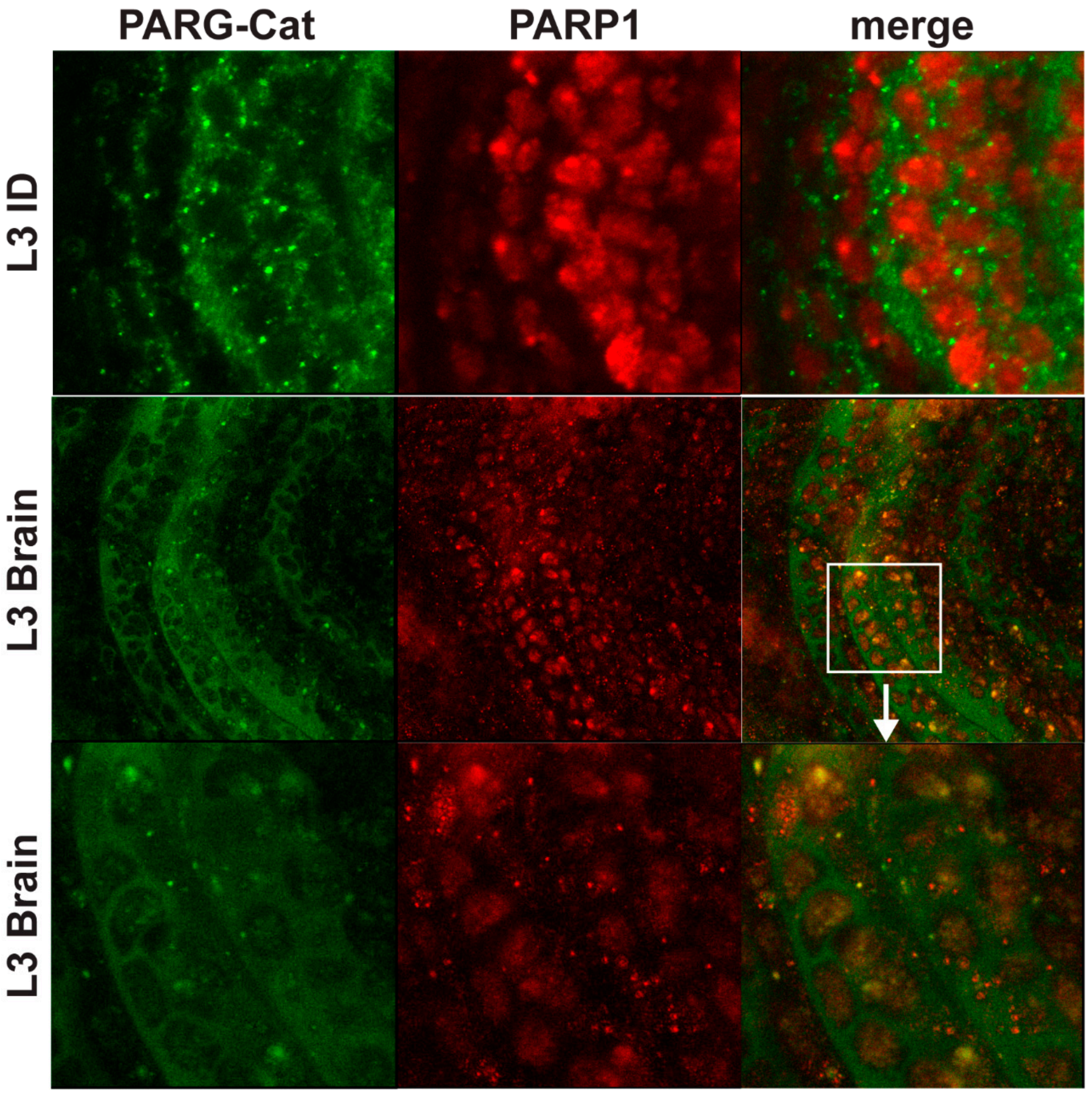

Supplemental Figure S6. PARG-Cat Localization in Diploid Tissues. Representative images showing the localization of PARG-Cat (left panels) and PARP1 localization (middle panels) in diploid tissues, including third instar larval imaginal discs and the brain.

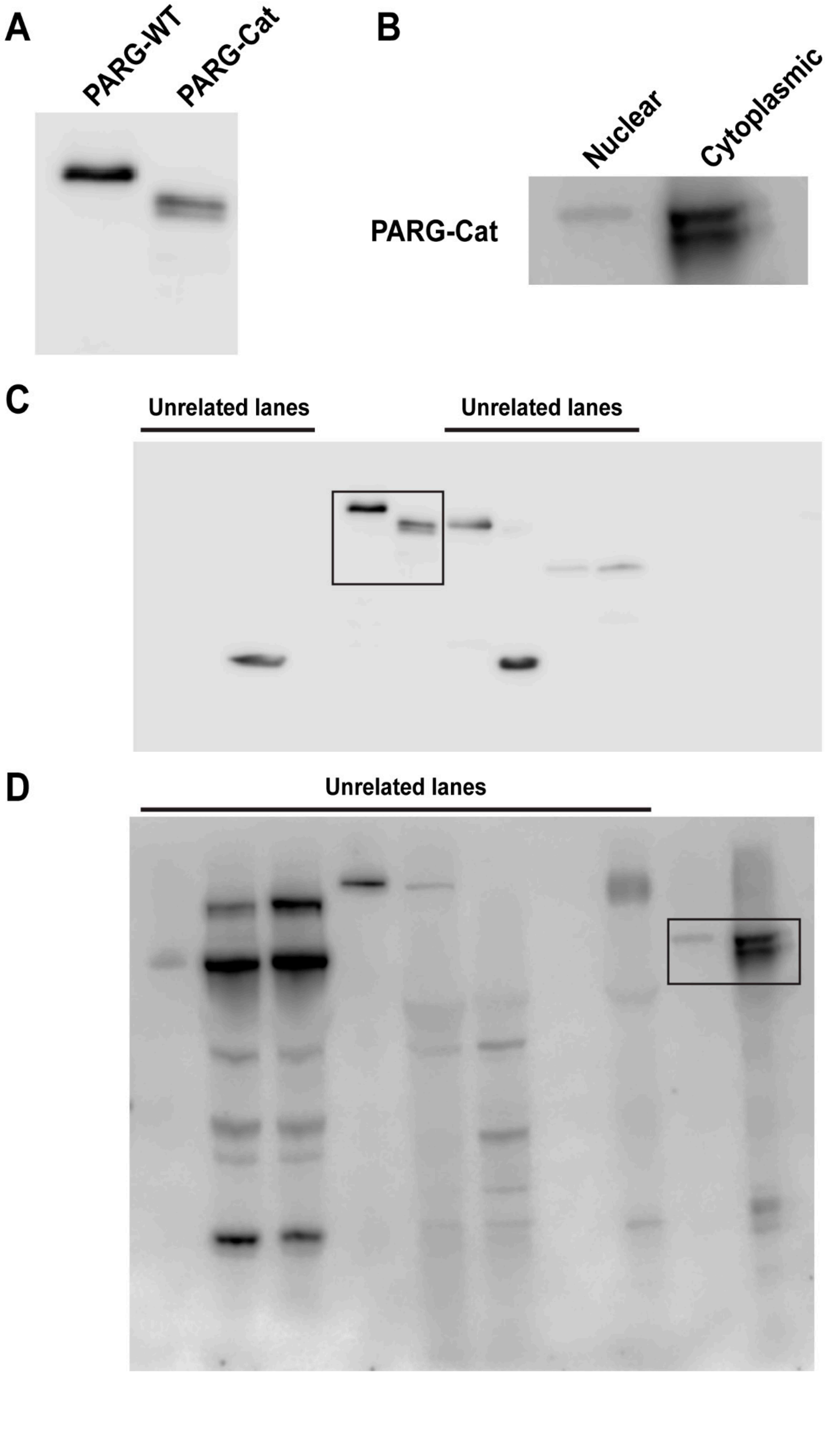
A
PARG-WT
PARG-Cat
B
Nuclear
Cytoplasmic
PARG-Cat
C
Unrelated lanes
Unrelated lanes
D
Unrelated lanes

Supplemental Figure S7. Only the Higher Molecular Weight Form of PARG-Cat Is Detected in the Nucleus. A) Western blot of GFP-tagged PARG-WT and PARG-Cat. PARG-Cat appears as two distinct bands, with the lower band corresponding to its predicted molecular weight. **B)** Western blot analysis of GFP-tagged PARG-Cat following subcellular fractionation. The left and right lanes correspond to the nuclear and cytoplasmic fractions, respectively. Only the higher molecular weight form of PARG-Cat is detected in the nuclear fraction, suggesting that this post-translationally modified form is required for nuclear retention. **C-D)** Uncropped western blot for panel A (**C**) or panel B (**D**). The black boxes indicate the regions shown in the cropped version. The other lanes correspond to samples unrelated to this study.

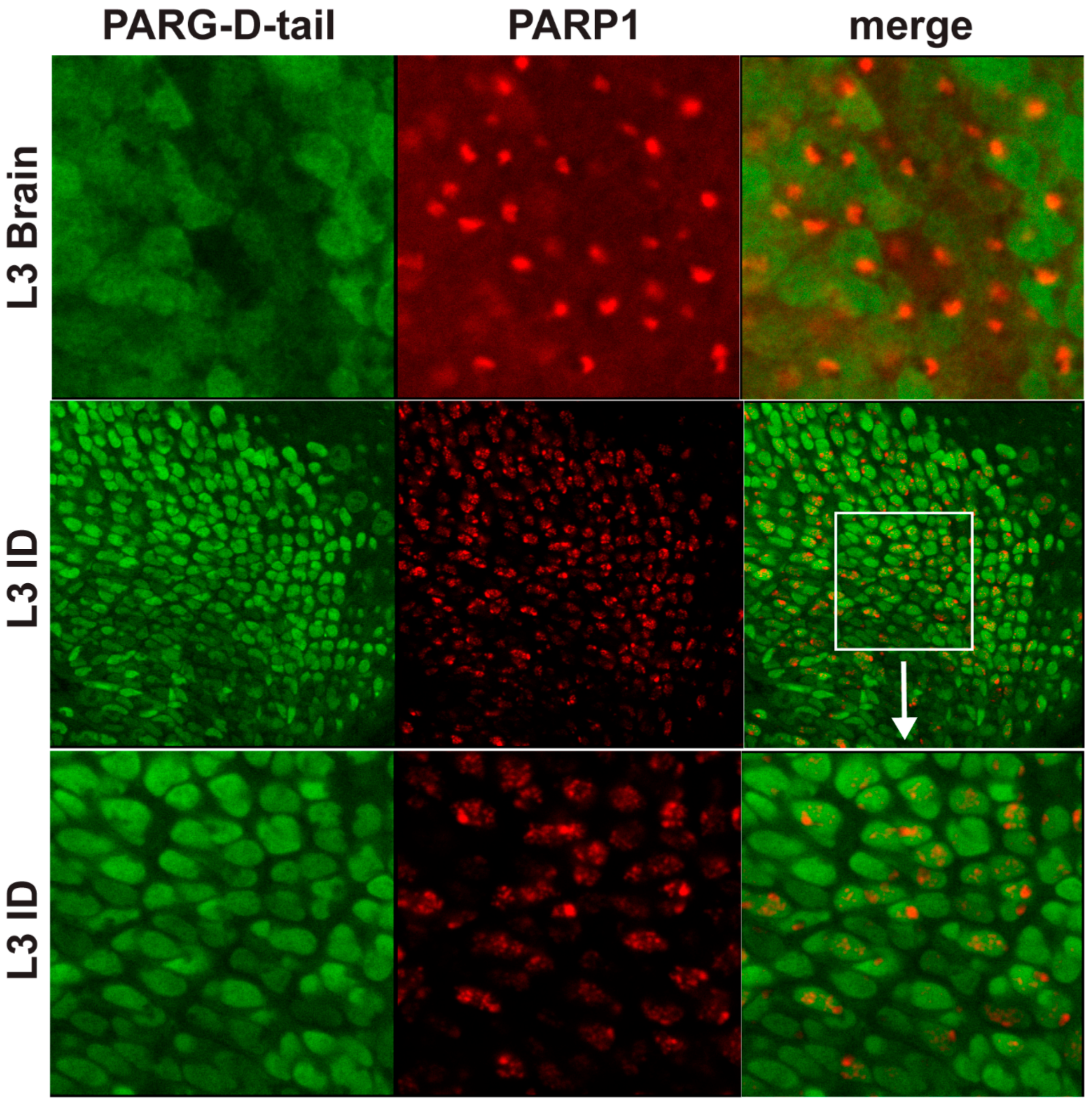

Supplemental Figure S8. PARG-D-tail Localization in Diploid Tissues. Representative images showing the localization of PARG-D-tail (left panels) and PARP1 localization (middle panels) in diploid tissues, including third instar larval imaginal discs and the brain.

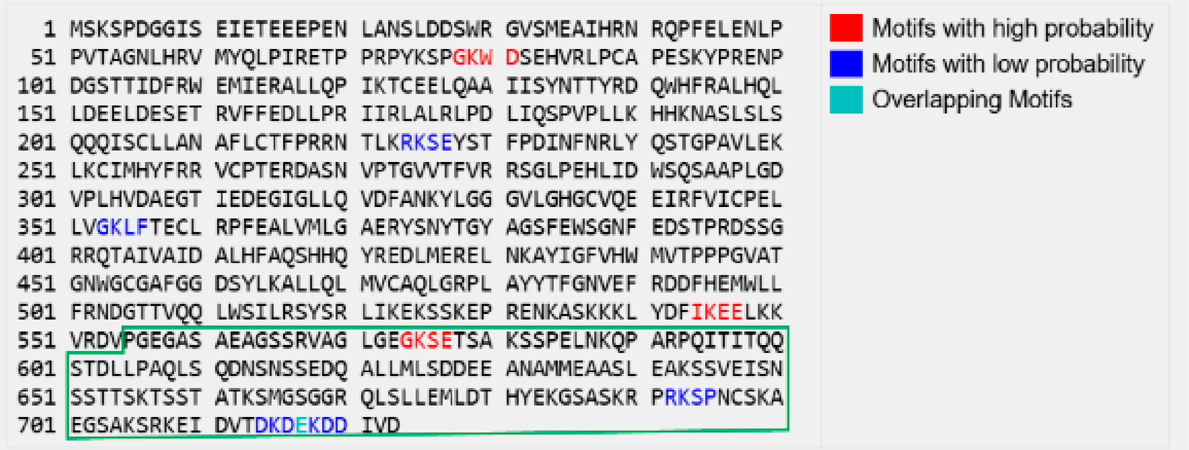

No.	Pos.	Group	Score	No.	Pos.	Group	Score
1	K545	KLYDF **IKEE** LKKVR	0.94	5	K715	EIDVT **DKDE** KDDIV	0.5
2	K575	AGLGE **GKSE** TSAKS	0.67	6	K225	RNTLK **RKSE** YSTFP	0.44
3	K79	PYKSP **GKWD** SEHVR	0.67	7	K693	ASKRP **RKSP** NCSKA	0.34
4	K718	VTDKD **EKDD** IVD	0.5	8	K354	PELLV **GKLF** TECLR	0.32

Supplemental Figure S9. Predicted PARG SUMOylation Motifs are Located on Catalytic Domain and on the Disordered Tail Region. Predicted PARG SUMOylation motifs by SUMOplot. The top panel exhibits Drosophila PARG protein sequence with the green box highlighting the C-terminal region. Motifs with high and low probability are highlighted in red and blue, respectively. The lower panel exhibits the score for each motif.

Supplemental Movie S1 legend. **The Disordered Tails of Human and Drosophila PARG Are Structurally Distinct**. This movie shows the aligned structures of human PARG (hPARG) and Drosophila PARG (dPARG) in rotation, highlighting their structural differences. The disordered tail and the catalytic domain of hPARG are labeled in yellow and pink, respectively, while the catalytic domain and the disordered tail of dPARG are labeled in blue and green, respectively.

Supplemental Table S1 legend. Mass Spectrometry Data. Comprehensive list of proteins identified in the mass spectrometry analysis of PARG interactors. Column A corresponds to the protein symbol, while column B provides the FlyBase ID. Column C indicates the molecular weight of each protein. Columns D to G display the spectral count of peptides identified in different conditions: PARG-WT (D), PARG-Cat (E), PARG-D-tail (F), or Control lines (G). Biological replicates are separated by a "|". Columns H, J, and L show the SAINT scores for PARG-WT (H), PARG-Cat (J), and PARG-D-tail (L), which reflect the confidence of protein-protein interactions. Columns I, K, and M report the SAINT False Discovery Rate (FDR) values for PARG-WT (I), PARG-Cat (K), and PARG-D-tail (M).

Supplemental Table S2 legend. Proteins Interacting with the Catalytic Domain. List of 289 proteins that significantly interact with the PARG catalytic domain; these were identified based on a SAINT score higher than 0.5 and a SAINT False Discovery Rate (FDR) lower than 15%. Column A corresponds to the protein symbol, while column B provides the FlyBase ID.

Supplemental Table S3 legend. Proteins Interacting with the Disordered Tail Region legend. List of 191 proteins that significantly interact with the disordered tail region of PARG; these were identified based on a SAINT score higher than 0.5 and a SAINT False Discovery Rate (FDR) lower than 15%. Column A corresponds to the protein symbol, while column B provides the FlyBase ID.
